# Safety and effectiveness of peficitinib (ASP015K) in patients with rheumatoid arthritis: interim data (22.7 months mean peficitinib treatment) from a long-term, open-label extension study in Japan, Korea, and Taiwan

**DOI:** 10.1186/s13075-020-2125-2

**Published:** 2020-03-12

**Authors:** Tsutomu Takeuchi, Yoshiya Tanaka, Sakae Tanaka, Atsushi Kawakami, Yeong-Wook Song, Yi-Hsing Chen, Mitsuhiro Rokuda, Hiroyuki Izutsu, Satoshi Ushijima, Yuichiro Kaneko, Yoshihiro Nakashima, Teruaki Shiomi, Emi Yamada

**Affiliations:** 1 Keio University School of Medicine, Tokyo, Japan; 2 University of Occupational and Environmental Health, Japan, Kitakyushu, Japan; 3 University of Tokyo, Tokyo, Japan; 4 Nagasaki University Graduate School of Biomedical Sciences, Nagasaki, Japan; 5 Seoul National University, Seoul National University Hospital, Seoul, South Korea; 6 Taichung Veterans General Hospital, Taichung, Taiwan; 7 Astellas Pharma, Inc., Tokyo, Japan

**Keywords:** Rheumatoid arthritis, Janus kinase, Peficitinib, Long-term extension study, ASP015K

## Abstract

**Background:**

Peficitinib (ASP015K), a novel oral Janus kinase inhibitor, has demonstrated efficacy and safety for the treatment of rheumatoid arthritis (RA) in randomized, controlled trials of up to 52 weeks’ duration. However, safety and effectiveness after long-term treatment have not been assessed.

**Methods:**

This was an interim analysis of an ongoing open-label, multicenter extension study in RA patients who completed phase 2b (RAJ1; 12 weeks) and phase 3 (RAJ3 and RAJ4; 52 weeks) peficitinib studies in Asia (mainly Japan). Eligible patients (*n* = 843) received oral peficitinib once daily (100 mg, or 50 mg for patients transferring from RAJ1). The peficitinib dose could be increased (up to 150 mg) or reduced (to 50 mg) at the discretion of the investigator. Efficacy variables assessed included American College of Rheumatology (ACR) response rates, ACR components, and disease activity score in 28 joints based on C-reactive protein (DAS28-CRP).

**Results:**

Results up to May 2018 are summarized. Mean peficitinib duration of exposure was 22.7 months and the maximum dose was 100 mg in most (66.5%) patients. ACR responses were maintained during the extension study, with ACR20/50/70 response rates of 71.6%, 52.1%, and 34.7% at week 0 and 78.9%, 61.4%, and 42.7% at end of treatment, respectively. ACR components and DAS28-CRP showed improvements from baselines of the preceding studies and continued to show improvements during the extension study. Treatment-emergent adverse events (TEAEs) were reported in 757/843 (89.8%) patients, the most common being nasopharyngitis (39.7%) and herpes zoster (11.7%). The majority of TEAEs were severity grade 1/2. Drug-related TEAEs leading to permanent study drug discontinuation occurred in 55/843 (6.5%) patients. Regarding AEs of special interest, the incidence per 100 patient-years of serious infections was 2.3 (95% CI 1.6 – 3.1), herpes zoster-related disease 6.8 (95% CI, 5.6 – 8.3), and malignancies 1.1 (95% CI, 0.7 – 1.8). One death from diffuse large B cell lymphoma during the study and one death from uterine sarcoma after the study were considered probably and possibly related to study drug, respectively.

**Conclusions:**

The effectiveness of peficitinib was maintained or improved during long-term administration and treatment up to 6 years was well tolerated in Asian patients with RA.

**Trial registration:**

ClinicalTrials.gov, NCT01638013, registered retrospectively 11 July 2012.

## Introduction

Rheumatoid arthritis (RA) is a chronic inflammatory disease characterized by persistent joint inflammation. Without adequate treatment, it can progress to joint deformity and functional impairment [1]. Current treatment of RA is based on disease-modifying antirheumatic drugs (DMARDs), usually starting with methotrexate (MTX), and biologic agents such as tumor necrosis factor-alpha (TNF-α) inhibitors in patients not responding to conventional DMARDs [2, 3]. The Janus kinase (JAK) family of non-receptor protein tyrosine kinases (JAK1, JAK2, JAK3, and tyrosine kinase 2 [TYK2]) plays a crucial role in multiple cytokine receptor signaling pathways [4] and is a promising target for RA treatment in patients with insufficient response to DMARDs and biologic agents. JAK inhibitors available for the treatment of patients with RA include tofacitinib (a pan-JAK inhibitor approved in the USA, Europe, and Asia [5–8]), baricitinib (a JAK1 and JAK2 selective inhibitor approved in the USA, Europe and Asia [9–12]), and more recently peficitinib (ASP015K; approved in Japan).

Peficitinib is an orally bioavailable, once-daily JAK inhibitor, developed for the treatment of patients with RA (including the prevention of structural injury of joints) who have had an inadequate response to DMARDs, including MTX. Peficitinib is a pan-JAK inhibitor that inhibits JAK1, JAK2, JAK3, and TYK2 [13]. Compared with other JAK inhibitors, peficitinib is moderately selective for JAK3 (over JAK1, JAK2, and TYK2) [4, 14]. Less potent inhibition of JAK2 may explain why reduced hemoglobin levels, potentially attributable to JAK2 inhibition, have not been observed with peficitinib [15, 16].

In a phase 2b, randomized, double-blind, placebo-controlled trial (RAJ1; ClinicalTrials.gov identifier, NCT01649999), peficitinib monotherapy showed efficacy and an acceptable safety profile in Japanese patients with moderate-to-severe RA after 12 weeks [15]. In two phase 3, randomized, double-blind, placebo-controlled trials, peficitinib treatment for 52 weeks showed significantly improved efficacy compared with placebo and an acceptable safety profile in patients with RA who had an inadequate response to DMARDs (RAJ3 study in Japan, Korea, and Taiwan; ClinicalTrials.gov identifier, NCT02308163 [17]) or MTX (RAJ4 study in Japan; ClinicalTrials.gov identifier, NCT02305849 [18]).

As peficitinib is a relatively novel compound, it is important to assess safety and effectiveness after long-term treatment. We present safety and efficacy data from a long-term, open-label extension study of patients who completed the phase 2b (RAJ1) or phase 3 (RAJ3 or RAJ4) peficitinib clinical trials.

## Methods

### Study design

This was an open-label, long-term extension study (“RAJ2”) conducted at 165 sites in Japan, nine sites in Korea, and nine sites in Taiwan (see Additional file 1: Study sites). The extension study commenced in June 2012 and, after marketing approval of peficitinib in Japan in March 2019, continues as a post-marketing clinical study in Japan, and a clinical study in Korea and Taiwan. Safety and efficacy data are presented at the cut-off date of 31 May 2018. The duration of study treatment, therefore, varied between patients.

**Fig. 1:**
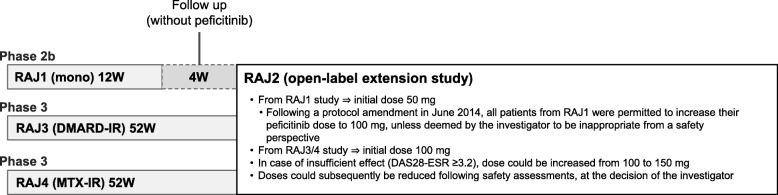
Design of the extension study

Enrolled patients with RA had completed one of three peficitinib clinical trials (Fig. 1): (1) “RAJ1,” a phase 2b trial in which patients received peficitinib monotherapy or placebo for 12 weeks with a 4-week follow up (without peficitinib treatment) [15]; (2) “RAJ3,” a phase 3 trial in which patients with an inadequate response to DMARDs (DMARD-IR) received peficitinib for 52 weeks (patients in the etanercept reference group of RAJ3 were not included in the extension study) [17]; and (3) “RAJ4,” a phase 3 trial in which patients with an inadequate response to MTX (MTX-IR) received peficitinib for 52 weeks [18].

Patients eligible for the extension study received oral peficitinib (50 mg, 100 mg, or 150 mg) once daily after breakfast. The starting dose was 100 mg (patients from RAJ3 and RAJ4) or 50 mg (patients from RAJ1). Following a protocol amendment in June 2014, all patients from RAJ1 were permitted to increase their peficitinib dose to 100 mg, unless deemed by the investigator to be inappropriate from a safety perspective. The dose could be increased following a lack of clinical response (DAS28-ESR ≥ 3.2 after 4 weeks of peficitinib treatment) and no potential safety concerns, as pre-specified in the protocol; the dose could also be reduced in line with safety assessments, as judged by the investigator. Treatment suspension (temporary discontinuation < 7 days), interruption (temporary discontinuation > 7 days and < 4 consecutive weeks, up to two per year separated by ≥ 16 weeks), or discontinuation during the extension study were at the discretion of the investigator and based on pre-specified criteria. In cases of suspension or interruption, peficitinib administration could be resumed if a retest confirmed that the suspension/interruption criteria were no longer met and the investigator deemed resumption of treatment to be in the patient’s best interests. Patients who discontinued early from the study were given the necessary tests (scheduled for the end of study) within 2 days after taking the final dose of the study drug, where possible. The reason for the discontinuation was recorded.

### Patients

Eligible patients with RA had completed study treatment in the preceding trials, as specified in the protocol, and completed assessments at week 16 (RAJ1 study) or week 52 (RAJ3 and RAJ4 studies). The baseline of this extension study (week 0) coincided with RAJ1 week 16 or RAJ3 and RAJ4 week 52.

Patients were excluded if they had any condition that would make them unsuitable for the study, as judged by the investigator. Patients were also excluded if they had taken, between the end of the preceding study and the start of the extension study: RAJ1, biologic DMARDs (etanercept, adalimumab, golimumab, infliximab, tocilizumab, abatacept, rituximab), nonbiologic DMARDs (MTX, salazosulfapyridine, gold, D-penicillamine, leflunomide, lobenzarit, actarit, tacrolimus, mizoribine, bucillamine, iguratimod, tofacitinib), or other RA drugs (e.g., cyclosporine, cyclophosphamide, azathioprine, minomycin); RAJ3 and RAJ4, prohibited concomitant medication/therapy (see below).

### Concomitant medications

The following concomitant medications (with dose adjustments) were permitted: NSAIDs, oral morphine (≤ 30 mg/day or equivalent of other opioid analgesics), and acetaminophen. If concurrent MTX was administered, the concomitant use of folic acid (maximum of 10 mg/week) was considered, where possible.

The following medications were prohibited during the extension study treatment period: biologic DMARDs (etanercept, anakinra, adalimumab, golimumab, infliximab, tocilizumab, abatacept, rituximab, certolizumab pegol, denosumab, sarilumab) and nonbiologic DMARDs according to preceding study (RAJ1: MTX, salazosulfapyridine, gold, D-penicillamine, leflunomide, lobenzarit, actarit, tacrolimus, mizoribine, bucillamine, iguratimod, tofacitinib, baricitinib; RAJ3: all except those used concomitantly during RAJ3; RAJ4: salazosulfapyridine, gold, D-penicillamine, leflunomide, lobenzarit, actarit, tacrolimus, mizoribine, bucillamine, iguratimod, tofacitinib, baricitinib).

The daily dose (prednisolone equivalent) of any oral corticosteroid was prohibited from exceeding the amount used at the initiation of studies RAJ1, RAJ3, or RAJ4.

### Outcomes

#### Efficacy endpoints

Efficacy endpoints were assessed in the overall population and in patients grouped according to their preceding study (RAJ1, RAJ3, or RAJ4): American College of Rheumatology (ACR)20/50/70 response rates, changes from the baselines of the preceding studies in ACR components, tender joint count at 68 joints (TJC68), swollen joint count at 66 joints (SJC66), Physician’s Global Assessment of Disease Activity (PGA), Subject’s Global Assessment of Disease Activity (SGA), Subject’s Global Assessment of Pain (SGAP), Health Assessment Questionnaire – Disability Index (HAQ-DI), change from the baselines of the preceding studies in 28-joint Disease Activity Score based on C-reactive protein (DAS28-CRP), proportion of patients achieving DAS28-CRP score < 2.6 (disease remission), and ACR20 response rate according to maximum peficitinib dose (50 mg, 100 mg, 150 mg).

#### Safety

The following safety assessments were reported during the overall period (from start of initial dosing in preceding studies): treatment-emergent adverse events (TEAEs), defined as any adverse event (AE) that started or worsened in severity after initial dose of study drug in the extension study until the end of the final observation, and TEAEs or serious AEs (SAEs) leading to permanent discontinuation. AEs of special interest—serious infections, herpes zoster-related disease, malignancies, and thromboembolic events (ad hoc analysis: first deep vein thrombosis [DVT] and pulmonary embolism [PE] identified using the MedDRA embolic and thrombotic SMQ preferred terms restricted to the respiratory, thoracic, mediastinal, and vascular disorder System Organ Classes)—were reported as incidence per 100 patient-years (95% confidence interval [CI]). Mean changes in laboratory measures, including hematology, lipoprotein, creatinine, creatine kinase, alanine aminotransferase (ALT), and aspartate aminotransferase (AST) were measured.

### Patient populations

The safety analysis set (SAF) included all patients who received at least one dose of study treatment. The full analysis set (FAS), used for efficacy analyses, included all patients who received at least one dose of study treatment and had measurements for any of the efficacy endpoints.

### Statistical analysis

Efficacy results were summarized by sample size, mean, standard deviation, minimum, median and maximum by time point for continuous variables, and frequency and percentage for categorical variables.

In relation to TEAEs, any unrecovered event that first occurred during the preceding studies (RAJ1, RAJ3, and RAJ4) was considered to be a medical condition and was not considered to be a TEAE in this extension study.

#### Missing data

The last observation carried forward (LOCF) method was used for ACR components, DAS28-CRP, and safety variables at the end of treatment (EOT). All outliers were included in the analysis.

### Ethics

This study was conducted in accordance with Good Clinical Practice, the International Council on Harmonization (ICH) of Technical Requirements for Registration of Pharmaceuticals for Human Use guidelines, and local laws and regulations. The protocol and amendments were approved by an Institutional Review Board (IRB) at each study site, and safety data were reviewed by the independent Data and Safety Monitoring Board. Each patient provided written informed consent prior to treatment initiation.

## Results

Data have been reported from the preceding phase 2b RAJ1 study [15], and the phase 3 RAJ3 [17] and RAJ4 [18] studies.

### Patient populations

**Fig. 2:**
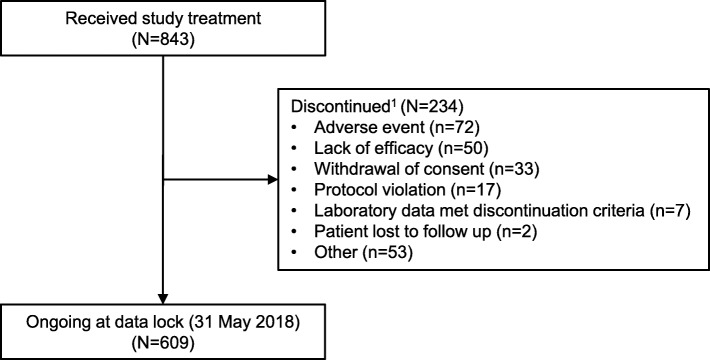
Patient flow through the extension study. ^1^Discontinued during overall period: discontinued at any time from start of initial dosing of study drug through the last dose day in the overall period

In total, 873 patients were screened for inclusion in the extension study. Of these, 843 patients received peficitinib treatment (Fig. 2): 201 patients from RAJ1, 225 from RAJ3, and 417 patients from RAJ4 (Additional file 1: Fig. S1). A total of 234 (27.8%) patients discontinued study treatment, mainly due to AEs (8.5%), other (6.3%), and lack of efficacy (5.9%) (Fig. 2). At the cut-off date for the present analysis (31 May 2018), 609 (72.2%) patients were still receiving study treatment (Fig. 2). All 843 patients who received treatment were included in the SAF, and 837 patients were included in the FAS.

### Demographics and characteristics at the start of the extension study

Most patients were female (619/843, 73.4%) and Japanese (806, 95.6%), with a mean age of 55.7 years (Table 1). The mean duration of RA was 6.2 years (SD 5.6). Concomitant MTX treatment was used by 65.1% of patients (Table 1).

**Table 1:** Patient demographics and characteristics at baseline of extension study (SAF)

	Total (*N* = 843)
Age, years	55.7 (11.9)
Female, *n* (%)	619 (73.4)
Study region, *n* (%)	
Japan	806 (95.6)
Korea	19 (2.3)
Taiwan	18 (2.1)
RA duration at baseline of preceding study, years^1^	6.2 (5.6)
Patients in prednisolone dose category, *n* (%)	
None	432 (51.2)
Average 0 – 5 mg/day	341 (40.5)
Average > 5 mg/day	70 (8.3)
Patients receiving concomitant DMARD, *n* (%)	
None	234 (27.8)
MTX	549 (65.1)
DMARD except for MTX	60 (7.1)
Maximum MTX dose for overall period, mg/week	9.7 (3.2)
Tender joint count at 68 joints^2^	5.1 (7.5)
Swollen joint count at 66 joints^2^	4.0 (5.3)
HAQ-DI score^3^	0.59 (0.58)
CRP, mg/dL^4^	0.87 (1.51)
ESR, mm/h^4^	28.4 (22.6)
DAS28-CRP^2^	3.03 (1.46)
DAS28-ESR^2^	3.63 (1.58)
CDAI score^2^	11.59 (11.83)
SDAI score^2^	12.47 (12.69)

Data are expressed as mean (SD) unless otherwise stated

^1^Duration of RA was calculated as (date of screening visit of preceding study – onset date of RA + 1)/365.25

^2^Higher scores indicate greater levels of disease activity

^3^Possible HAQ-DI scores range 0 – 3, with higher scores indicating greater disability

^4^Higher CRP and ESR values indicate greater inflammation

*CDAI* Clinical Disease Activity Index, *CRP* C-reactive protein, *DAS* Disease Activity Score, *DMARD* disease-modifying antirheumatic drug, *ESR* erythrocyte sedimentation rate, *HAQ-DI* Health Assessment Questionnaire – Disability Index, *MTX* methotrexate, *RA* rheumatoid arthritis, *SAF* safety analysis set, *SD* standard deviation, *SDAI* Simplified Disease Activity Index

There were some differences in characteristics at baseline of the extension study according to the preceding study (Additional file 1: Table S1): all patients from RAJ1 and RAJ4 were Japanese, whereas the RAJ3 study included patients from Japan (83.6%), Korea (8.4%), and Taiwan (8.0%); the mean duration of RA was higher in patients from RAJ1 (7.3 years, SD 6.1) and RAJ3 (8.7 years, SD 7.2) than RAJ4 (4.3 years, SD 3.0); concomitant MTX treatment was used by 0%, 60.4%, and 99.0% of patients in the RAJ1, RAJ3, and RAJ4 studies, respectively.

### Treatment exposure

The mean duration of peficitinib exposure was 22.7 months (maximum 70.7 months) (Table 2). The mean duration was longer in patients from RAJ1 (41.6 months) than RAJ3 (17.9 months) and RAJ4 (16.2 months) (Additional file 1: Table S2); this reflected the earlier date of first treatment initiation in the RAJ1 study (22 March 2012), compared with RAJ3 (8 September 2014) and RAJ4 (29 August 2014).

**Table 2:** Peficitinib treatment exposure and changes in peficitinib dose during the overall period (SAF)

	Total (*N* = 843)
Duration of peficitinib exposure, months^1^	
Mean (SD)	22.7 (17.4)
Max	70.7
Median	18.2
Min	0.1
Duration of initial peficitinib dose, months^2^	
Mean (SD)	12.1 (11.7)
Max	70.7
Min	0.1
Treatment compliance rate (%)^3^	
Mean (SD)	97.0 (3.5)
Dose increase, *n* (%)	
No	489 (58.0)
Yes	354 (42.0)
1 dose increase	285 (33.8)
2 dose increases	63 (7.5)
≥ 3 dose increases	6 (0.7)
Dose decrease, *n* (%)	
No	802 (95.1)
Yes	41 (4.9)
1 dose decrease	38 (4.5)
2 dose decreases	3 (0.4)
≥ 3 dose decreases	0
Maximum peficitinib dose, *n* (%)	
50 mg	39 (4.6)
100 mg	561 (66.5)
150 mg	243 (28.8)

^1^Duration of exposure for overall period (days) was calculated as: date of the last dose of study drug – date of initial dose of study drug + 1

^2^Duration from first peficitinib taken (50 mg for patients from RAJ1, 100 mg for patients from RAJ3 and RAJ4) up to first dose change was calculated

^3^Treatment compliance for overall period (%) was calculated as: 100 × (total number of tablets actually received in the overall period/total number of tablets planned to receive in the overall period)

*SAF* safety analysis set, *SD* standard deviation

During the overall period, the peficitinib dose was increased from the initial administered dose, or after a dose reduction, in 42.0% of all patients (Table 2). A higher proportion of patients from RAJ1 (80.6%) than RAJ3 (30.7%) and RAJ4 (29.5%) had an increase in peficitinib dose. This is because the initial administered dose for patients from RAJ1 entering RAJ2 was 50 mg but, following a protocol amendment in June 2014, all patients from RAJ1 were permitted to have a dose increase to 100 mg, unless safety findings deemed it inappropriate (Additional file 1: Table S2).

The maximum peficitinib dose was 100 mg for most patients: 66.5% of patients overall, and 71.1% and 71.2% of patients from RAJ3 and RAJ4, respectively (Table 2; Additional file 1: Table S2). In RAJ1, the maximum dose was 50 mg in 19.4% of patients, 100 mg in 51.7% of patients, and 150 mg in 28.9% of patients (Additional file 1: Table S2). Mean treatment compliance was 97.0% overall (range, 96.3–98.8% by preceding study) (Table 2; Additional file 1: Table S2).

### Efficacy

#### ACR response

**Fig. 3:**
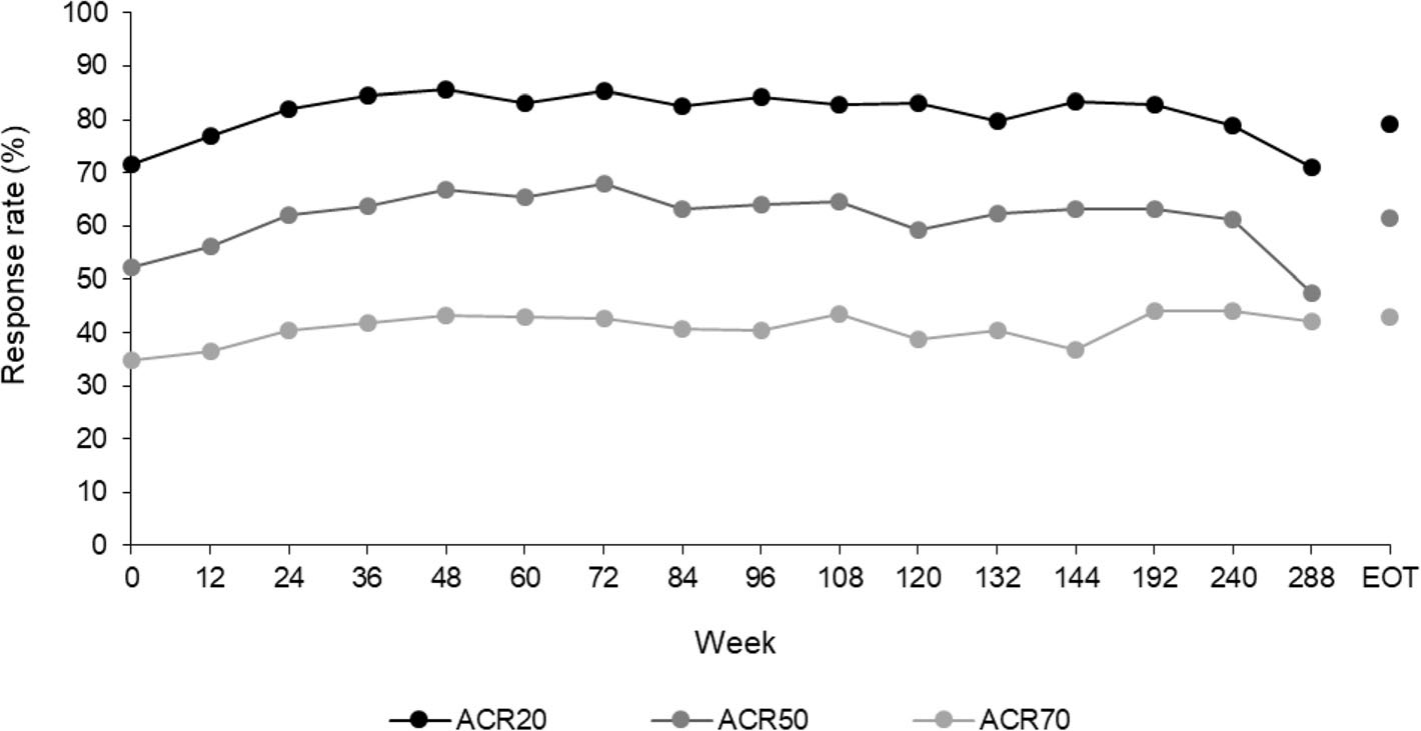
Response rates for ACR20, ACR50, and ACR70 over time (FAS)

In the overall population, the ACR20, ACR50, and ACR70 response rates were 71.6%, 52.1%, and 34.7%, respectively, at the baseline of this extension study (week 0). These rates were maintained during the extension study, and at the end of treatment were 78.9%, 61.4%, and 42.7%, respectively (Fig. 3). When patients were grouped according to their preceding study, the response rates were maintained in those patients from RAJ3 and RAJ4. In patients from the RAJ1 study, the response rates improved for the first 4 weeks and were then maintained during the extension study (Additional file 1: Fig. S2).

ACR components (TJC68, SJC66, SGAP, SGA, PGA, HAQ-DI) improved from the baselines of the preceding studies and continued to improve during the extension study (Additional file 1: Fig. S3).

#### ACR20

**Fig. 4:**
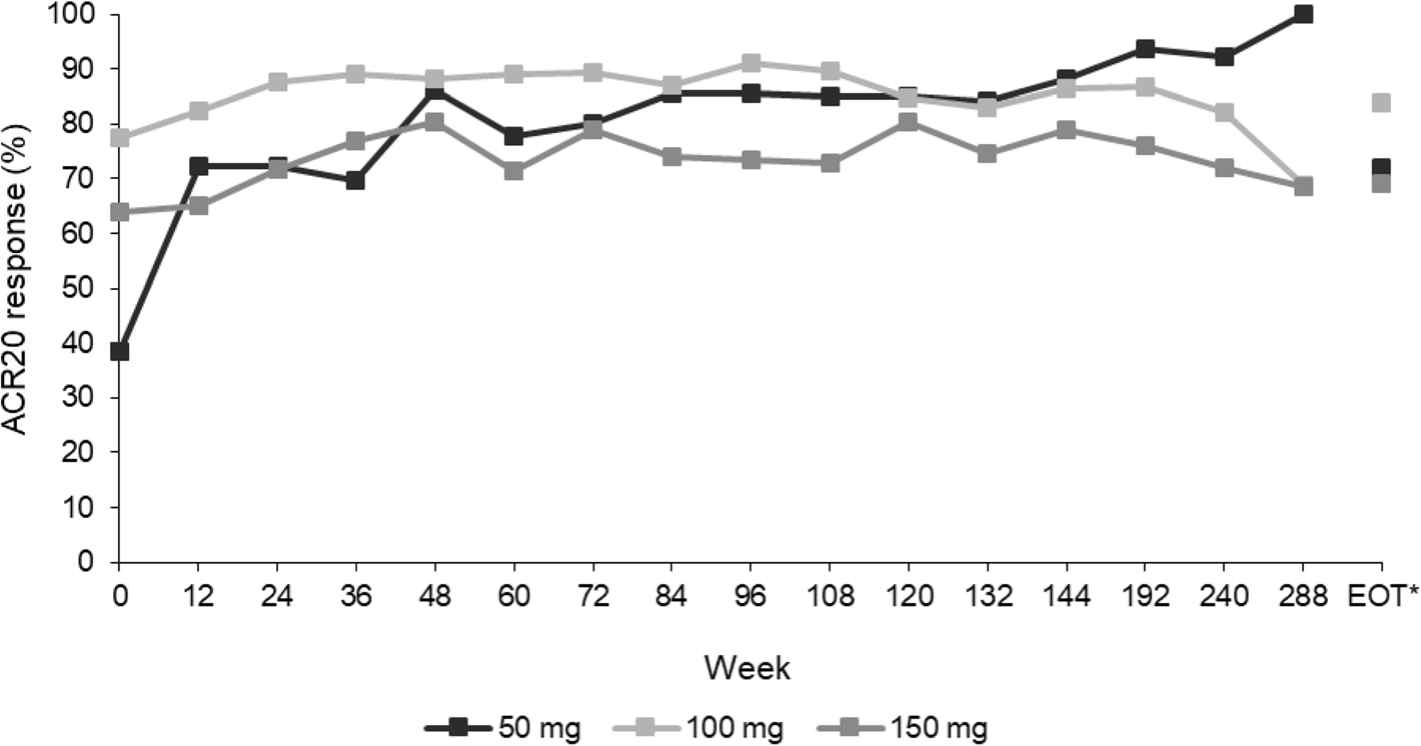
ACR20 response at each visit by maximum peficitinib dose level (FAS). *Includes LOCF

The ACR20 response rate was maintained during the extension study in patients with a maximum peficitinib dose of 100 or 150 mg. In patients with a maximum dose of 50 mg, the ACR20 response rate improved at the beginning of the extension study and was then maintained (Fig. 4).

#### DAS28-CRP

**Fig. 5:**
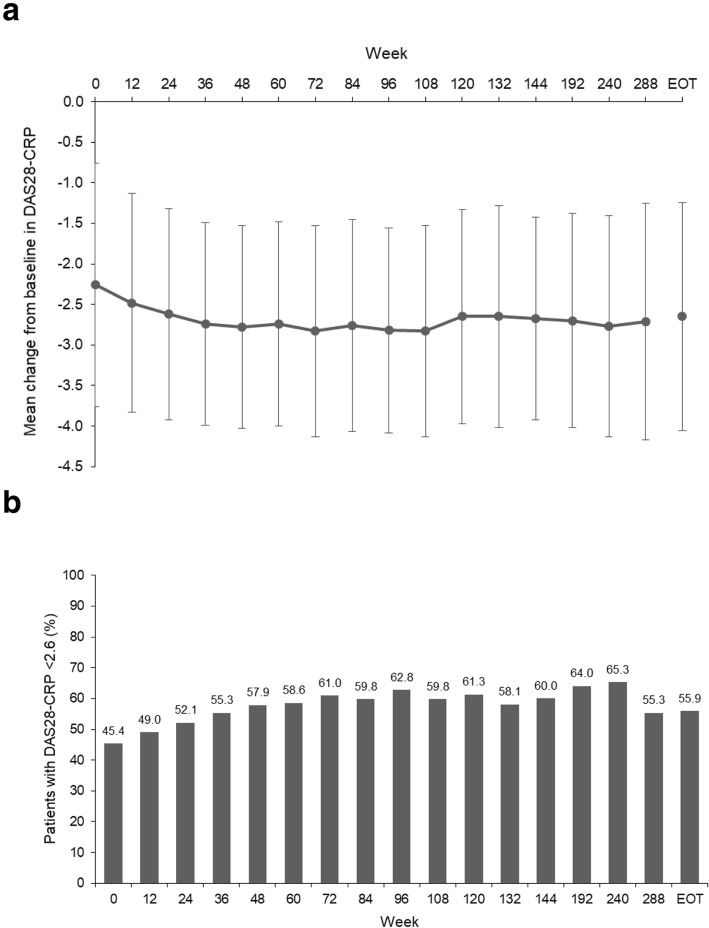
**a** Mean DAS28-CRP changes from baseline, **b** patient proportions achieving DAS28-CRP score < 2.6 (FAS)

The improvement in DAS28-CRP was maintained during the extension study (Fig. 5a): this was seen overall (Fig. 5a) and in patients from RAJ3 and RAJ4 (Additional file 1: Fig. S4a). In patients from the RAJ1 study, DAS28-CRP improved early in the extension study and was then maintained (Additional file 1: Fig. S4a). The proportion of patients achieving DAS28-CRP remission (score of < 2.6) was maintained during the extension study: this was seen overall (Fig. 5b) and in patients from RAJ3 and RAJ4 (Additional file 1: Fig. S4b). In patients from RAJ1, DAS28-CRP improved early in the extension study and was then maintained (Additional file 1: Fig. S4b).

### Safety

#### Adverse events

Of 843 patients in the SAF, TEAEs were reported in 757 (89.8%) patients, and SAEs in 138 (16.4%) patients (Table 3). TEAEs and SAEs leading to permanent discontinuation of study drug were reported in 87 (10.3%) and 50 (5.9%) patients, respectively. The majority of TEAEs were grade 1 or 2 in severity. Table 4 summarizes TEAEs that occurred in ≥ 5% of patients. The most common were nasopharyngitis (39.7%), RA (12.5%), and herpes zoster (11.7%). Drug-related AEs were reported in 586 (69.5%) patients, and drug-related SAEs were reported in 76 (9.0%) patients. Drug-related TEAEs leading to permanent discontinuation of peficitinib were reported in 55 (6.5%) patients. The most frequent drug-related SAEs were seven cases of herpes zoster (0.8%) and six cases of pneumonia (0.7%).

**Table 3:** Overview of treatment-emergent adverse events (TEAEs) in the overall period (SAF)

	Total (*N* = 843) *n* (%)
All TEAEs	757 (89.8)
Drug-related^1^ TEAEs	586 (69.5)
Drug-related^1^ SAEs	76 (9.0)
≥ Grade 3 TEAE^2^	189 (22.4)
TEAEs leading to permanent discontinuation of study drug	
All	87 (10.3)
Drug-related^1^	55 (6.5)
SAEs	50 (5.9)
Drug-related^1^ SAEs	30 (3.6)

Treatment-emergent adverse events were defined as any AE that started or worsened in severity after initial dose of study drug in the extension study until the end of the final observation

^1^Possibly or probably related to study drug, as assessed by the investigator or records where relationship was missing

^2^National Cancer Institute Common Terminology Criteria for Adverse Events (NCI-CTCAE): grade 3, severe or medically significant; grade 4, life-threatening; grade 5, death related to AE

*AE* adverse event, *SAE* serious adverse event, *SAF* safety analysis set

**Table 4:** Treatment-emergent adverse events occurring in ≥ 5% of patients in the overall period (SAF)

	Total (*N* = 843) *n* (%)
Nasopharyngitis	335 (39.7)
Rheumatoid arthritis	105 (12.5)
Herpes zoster	99 (11.7)
Influenza	80 (9.5)
Bronchitis	68 (8.1)
Blood creatine phosphokinase increased	66 (7.8)
Contusion	59 (7.0)
Hypertension	57 (6.8)
Pharyngitis	56 (6.6)
Dental caries	51 (6.0)
Upper respiratory tract infection	51 (6.0)
Constipation	49 (5.8)
Cystitis	47 (5.6)
Gastroenteritis	47 (5.6)
Back pain	42 (5.0)
Cough	42 (5.0)

*SAF* safety analysis set

There was one death (0.1%) during the study, due to diffuse large B cell lymphoma, which was considered probably related to study drug, and there was one death after the end of the study, due to uterine sarcoma, which was considered possibly related to study drug (see Additional file 1: Case histories).

**Fig. 6:**
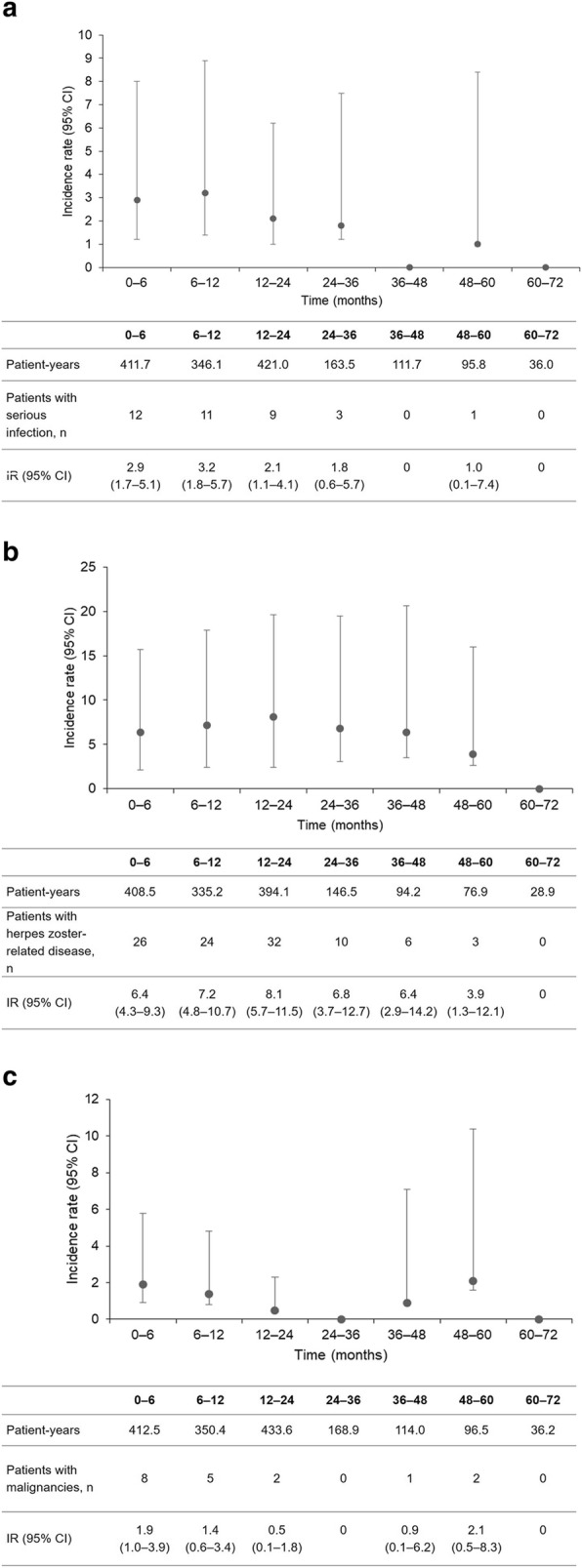
Adverse events of special interest per 100 patient-years during the overall period: **a** serious infections, **b** herpes zoster-related disease, **c** malignancies (SAF). Patient-years was calculated from initial dose up to first incidence of the event for patients who had at least one event, and from initial dose through follow up for patients who had no events; IR was calculated as (100 × number of patients with ≥ 1 incidence/total patient-years) *CI* confidence interval, *IR* incidence rate

Regarding AEs of special interest, the incidence per 100 patient-years was 2.3 (95% CI, 1.6–3.1) for serious infections, 6.8 (95% CI, 5.6–8.3) for herpes zoster-related disease, and 1.1 (95% CI, 0.7–1.8) for malignancies. Figure 6 summarizes the incidence of these events per 100 patient-years during the overall period. Peficitinib exposure was 1585.8, 1484.4, and 1612.0 patient-years for serious infections, herpes zoster, and malignancy (overall period in the total peficitinib population). In an ad hoc analysis of thromboembolic AEs, total peficitinib exposure was 1615.2 patient-years and incidence per 100 patient-years was 0.1 (95% CI, 0.0 – 0.5). There was one case of deep vein thrombosis (0.1%) and one case of pulmonary artery thrombosis (0.1%). In both cases, the study physician regarded the event as not related to peficitinib.

#### Laboratory measures

An increase from baseline (week 0) in creatine kinase and a decrease in lymphocytes was observed during the extension study (Table 5). There were no notable changes from baseline (week 0) in other laboratory parameters, including neutrophils, hemoglobin, platelets, low-density lipoprotein, high-density lipoprotein, creatinine, ALT, or AST (Table 5).

**Table 5:** Mean changes from baseline (week 0) in laboratory measurements (SAF)

	Mean (SD) at week 0	Mean (SD) change from baseline									
		Week 24	Week 48	Week 72	Week 96	Week 120	Week 144	Week 192	Week 240	Week 288	EOT
Absolute neutrophil count, 10^6^/L	4826.2 (1920.5)	−8.5 (1413.9)	−31.3 (1397.3)	−92.2 (1411.7)	46.2 (1564.6)	− 206.7 (1560.6)	− 342.5 (1681.6)	− 457.7 (1685.6)	− 370.5 (1518.7)	− 621.1 (1558.1)	− 115.7 (1694.4)
Hemoglobin, g/L	126.0 (14.3)	0.9 (7.6)	1.6 (8.7)	2.7 (10.1)	3.5 (10.0)	4.7 (10.2)	6.1 (11.2)	5.2 (11.4)	5.7 (11.5)	4.9 (9.3)	1.2 (9.9)
Lymphocytes, 10^6^/L	1429.8 (555.9)	−81.3 (382.1)	−151.1 (388.5)	− 195.6 (398.1)	− 270.2 (408.0)	− 310.3 (470.1)	− 434.2 (437.2)	− 468.5 (458.8)	− 480.0 (484.8)	− 647.4 (501.2)	− 216.4 (442.2)
Platelets, 10^9^/L	273.9 (71.0)	−7.1 (42.6)	−10.3 (44.9)	−13.4 (46.6)	−16.4 (53.5)	−17.3 (55.7)	−29.1 (57.6)	−24.7 (63.2)	−26.9 (61.0)	−17.9 (62.5)	2.2 (50.1)
LDL-C, mmol/L	3.115 (0.858)	0.052 (0.561)	0.093 (0.631)	0.102 (0.673)	0.085 (0.708)	0.065 (0.811)	0.114 (0.854)	0.186 (0.910)	0.183 (0.772)	0.378 (0.877)	0.055 (0.734)
HDL-C, mmol/L	1.889 (0.547)	0.074 (0.316)	0.110 (0.294)	0.143 (0.335)	0.180 (0.340)	0.273 (0.334)	0.382 (0.334)	0.327 (0.344)	0.359 (0.298)	0.486 (0.394)	0.116 (0.352)
Creatinine, μmol/L	58.17 (14.55)	1.09 (6.38)	2.37 (6.42)	2.91 (6.88)	4.03 (7.05)	5.16 (7.66)	8.15 (7.21)	7.93 (6.83)	8.42 (7.07)	7.96 (9.11)	2.16 (8.19)
Creatine kinase, U/L	131.2 (124.8)	3.0 (109.8)	13.7 (120.4)	20.5 (151.8)	30.5 (105.5)	41.0 (86.4)	69.7 (66.4)	82.4 (88.3)	77.3 (84.6)	93.1 (74.7)	14.2 (132.5)
ALT, U/L	22.9 (16.5)	0.7 (18.4)	0.9 (14.0)	1.3 (16.1)	2.3 (15.7)	2.0 (13.1)	5.9 (14.3)	3.9 (10.1)	5.9 (12.7)	5.7 (9.4)	0.9 (16.4)
AST, U/L	27.1 (11.9)	0.7 (15.3)	1.3 (10.8)	1.7 (11.5)	2.2 (10.8)	2.0 (9.7)	5.0 (9.1)	4.0 (8.0)	5.2 (8.1)	6.2 (7.1)	1.3 (12.6)

EOT assessments and tests were to be performed promptly after the end of peficitinib administration; if peficitinib administration was terminated early, these assessments and tests were to be performed within 2 days of the last dose of peficitinib, if possible. As the timing of rollover from the previous study, and consequently the completion of the present trial, varied between individual patients, laboratory data at EOT were averaged regardless of the exact date of the EOT visit. *ALT* alanine aminotransferase, *AST* aspartate aminotransferase, *EOT* end of treatment, *HDL-C* high-density lipoprotein cholesterol, *LDL-C* low-density lipoprotein cholesterol, *SAF* safety analysis set, *SD* standard deviation, *Wk* week

## Discussion

Peficitinib, a novel oral JAK inhibitor, previously demonstrated clinical safety and efficacy in patients with RA in a 12-week phase 2b (RAJ1) trial and two 52-week phase 3 (RAJ3 and RAJ4) trials. In this open-label extension study of patients with RA who completed these three trials, we found that peficitinib treatment was associated with ACR20/50/70 responses that were maintained during long-term treatment. In patients with a maximum peficitinib dose of 100 mg or 150 mg, the ACR20 response rate was maintained and in patients with a maximum peficitinib dose of 50 mg, the ACR20 response rate improved and was then maintained during the extension study.

Of note, patients from the RAJ1 study had a lower ACR20 response rate at baseline (week 0) of the extension study than patients from RAJ3 and RAJ4 (Additional file 1: Fig. S2). This was not surprising as some patients enrolled in RAJ1 had received a low dose (25 or 50 mg) of peficitinib, or no peficitinib in the case of the placebo arm, in addition to others who had received peficitinib 100 mg or 150 mg; furthermore, the treatment period was 12 weeks, followed by a 4-week follow-up period (without peficitinib treatment) before enrollment in the extension study. In contrast, patients from RAJ3 and RAJ4 had all received at least 24 weeks’ treatment with peficitinib 100 mg or 150 mg, after which they were immediately enrolled in the extension study.

Further evidence of favorable clinical responses was provided by measurements of ACR components and DAS28-CRP. ACR components (TJC68, SJC66, SGAP, SGA, PGA, HAQ-DI) and DAS28-CRP improved from the baselines of the preceding studies and continued to improve during the extension study.

The safety analysis indicated that peficitinib treatment was well tolerated over a period of up to 6 years. Rates of AEs of special interest (serious infections, herpes zoster-related disease and malignancies) were consistent with studies of other JAK inhibitors. For tofacitinib and baricitinib, respectively, the reported rates of serious infections per 100 patient-years were 2.7 (including any event requiring hospitalization or parenteral antimicrobial therapy, or otherwise meeting SAE criteria) and 2.9 (including any event meeting ICH E2A criteria), respectively [19, 20]. Rates of herpes zoster-related disease for tofacitinib and baricitinib were 9.2 (in patients from Japan and Korea [21]) and 6.5 (in patients from Japan [22]), and 0.9 and 0.8 for malignancies, respectively [19, 20]. It should be noted, however, that drug exposure was higher in the tofacitinib (19,406 patient-years [20]) and baricitinib (6637 patient-years [19]) studies. Further follow up is required to assess the significance of the observed increases in creatine kinase and decreases in lymphocytes.

A concern has been raised regarding the use of high doses of tofacitinib to treat patients with RA, due to a safety signal for pulmonary embolism and increased mortality that has emerged during a post-marketing trial [23]. As of the cut-off date, two thromboembolic AEs were reported during the peficitinib extension study, one pulmonary artery thrombosis (0.1%) and one deep vein thrombosis (0.1%), but these AEs were considered by the study physician to be unrelated to peficitinib.

In the preceding phase 3 clinical trials, peficitinib (100 mg/day and 150 mg/day) provided superior efficacy to placebo, with clinical improvements observed by week 12 in patients with DMARD-IR (RAJ3) or MTX-IR (RAJ4). Overall, the interim analysis of this extension study also suggests that peficitinib may be a well-tolerated, effective treatment option for patients with RA in the longer term.

The long treatment period (mean duration of peficitinib exposure 22.7 months; maximum: 70.7 months) and the large number of treated patients (*n* = 843) are notable strengths of this study. This complements the long-term safety and efficacy data already available for other JAK inhibitors, including up to 8.5 years with tofacitinib [20, 24] and up to 2.5 years with baricitinib [25]. In addition, the inclusion of patients with varying treatment regimens (such as DMARD combinations, including MTX, as well as monotherapy) and the flexible study design, allowing individualized treatment of RA patients, is likely to be representative of routine medical practice. For example, the study allowed a dose increase in cases of DAS28-CRP > 3.2 and a dose reduction to 50 mg according to a patient’s condition or safety issues, which is similar to the way in which MTX is currently administered.

However, there are also a number of limitations associated with this extension study: patients’ experience of study treatment in the preceding studies may have influenced their decision to participate; the sample size was entirely dependent on the number of patients who completed the preceding studies; and there was no comparator group (placebo or active) during the extension. Furthermore, comparisons between peficitinib doses were limited because (1) patients were not randomized to different dose groups; (2) dose changes could be made (at the discretion of the investigator); and (3) patient disease states may have changed during the extension study. The patient population was drawn mainly from Japan (95.6%) and the data therefore lack global diversity. The Japanese population has a unique genetic, environmental, and medical background, which is known to influence the effectiveness and safety of biologic agents for RA [26]. This limits the generalizability of findings from this study to other populations.

At the cut-off date, 609 (72.2%) patients were still receiving peficitinib treatment and no new safety signals had been identified. Another phase 3 clinical trial is being conducted at multiple sites in China, Taiwan, and Korea to assess the safety and efficacy of 52 weeks’ peficitinib treatment in patients with RA who had an inadequate response to MTX (ClinicalTrials.gov identifier, NCT03660059, estimated completion date June 2020). This will further add to the evidence base for the efficacy and safety of peficitinib for RA in Asian patients.

## Conclusion

Administration of peficitinib for a mean duration of 22.7 months provided sustained improvements in ACR response rates, ACR core components, and DAS28-CRP. Peficitinib administration for up to 6 years was well tolerated and no new safety signals were identified. These results suggest that peficitinib may be an effective long-term treatment option for Asian patients with RA.

## Supplementary information

Additional file 1: **Table S1** Patient demographics and characteristics by preceding study at the extension study baseline (SAF). **Table S2** Peficitinib treatment exposure and changes in peficitinib dose during the overall period, by preceding study (SAF). **Fig. S1** Patient flow through the study, by preceding study. **Fig. S2** Response rates by preceding study: **a** ACR20, **b** ACR50, **c** ACR70 over time (FAS). **Fig. S3** Mean changes from the baselines of the preceding studies in **a** TJC68, **b** SJC66, **c** SGAP, **d** SGA, **e** PGA, and **f** HAQ-DI (FAS). **Fig. S4a** Mean changes from the baselines of the preceding studies in DAS28-CRP, by preceding study; **b** proportion of patients achieving DAS28-CRP < 2.6, by preceding study (FAS). **Study sites.Case histories of patients who died.**

## Data Availability

Researchers may request access to anonymized participant level data, trial level data and protocols from Astellas sponsored clinical trials at www.clinicalstudydatarequest.com. For the Astellas criteria on data sharing see: https://clinicalstudydatarequest.com/Study-Sponsors/Study-Sponsors-Astellas.aspx

## Abbreviations

ACR: American College of Rheumatology
ALT: Alanine aminotransferase
AST: Aspartate aminotransferase
CRP: C-reactive protein
DAS: Disease activity score
DMARD: Disease-modifying antirheumatic drug
EOT: End of treatment
FAS: Full analysis set
HDL-C: High-density lipoprotein cholesterol
HAQ-DI: Health Assessment Questionnaire – Disability Index
JAK: Janus kinase
LDL-C: Low-density lipoprotein cholesterol
LOCF: Last observation carried forward
MTX: Methotrexate
PGA: Physician’s Global Assessment of Disease Activity
RA: Rheumatoid arthritis
SAE: Serious adverse events
SD: Standard deviation
SGA: Subject’s Global Assessment of Disease Activity
SGAP: Subject’s Global Assessment of Pain
SJC: Swollen joint count
TEAE: Treatment-emergent adverse events
TJC: Tender joint count
TNF-α: Tumor necrosis factor-alpha
TYK: Tyrosine kinase
SAF: Safety analysis set

## References

[1] Cheung TT, McInnes IB (2017). Future therapeutic targets in rheumatoid arthritis?. Semin Immunopathol.

[2] Rubbert-Roth A, Finckh A (2009). Treatment options in patients with rheumatoid arthritis failing initial TNF inhibitor therapy: a critical review. Arthritis Res Ther.

[3] O’Dell JR (2004). Therapeutic strategies for rheumatoid arthritis. N Engl J Med.

[4] Hamaguchi H, Amano Y, Moritomo A, Shirakami S, Nakajima Y, Nakai K (2018). Discovery and structural characterization of peficitinib (ASP015K) as a novel and potent JAK inhibitor. Bioorg Med Chem.

[5] US Food and Drug Administration. FDA Approves Xeljanz. 2012. https://www.drugs.com/newdrugs/fda-approves-xeljanz-rheumatoid-arthritis-3558.html: Accessed 6 Aug 2019.

[6] Committee for Medicinal Products for Human Use (CHMP). Xeljanz - Assessment report. 2017. https://www.ema.europa.eu/documents/assessment-report/xeljanz-epar-public-assessment-report_en.pdf. Accessed 6 Aug 2019.

[7] Japan Ministry of Health Labour and Welfare. Xeljanz Tablets 5 mg report. 2013. https://www.pmda.go.jp/files/000153609.pdf. Accessed 6 Aug 2019.

[8] Pfizer’s rheumatoid arthritis drug adds indication for ulcerative colitis. Korea Biomed. Rev. 2018. http://www.koreabiomed.com/news/articleView.html?idxno=4678. Accessed 6 Aug 2019.

[9] US Food and Drug Administration. FDA approves Olumiant. 2018. https://www.drugs.com/newdrugs/fda-approves-olumiant-baricitinib-2-mg-adults-moderately-severely-active-rheumatoid-arthritis-4760.html: Accessed: 6 Aug 2019.

[10] Committee for Medicinal Products for Human Use (CHMP). Olumiant - Assessment report. 2016. https://www.ema.europa.eu/en/documents/assessment-report/olumiant-epar-public-assessment-report_en.pdf. Accessed 6 Aug 2019.

[11] Japan Ministry of Health Labour and Welfare. Report on the Deliberation Results: Olumiant Tablets 2 mg, 4 mg. 2017. http://www.pmda.go.jp/files/000226301.pdf. Accessed 6 Aug 2019.

[12] Korea approves Olumiant pills for treatment of rheumatoid arthritis. 2017. http://www.koreabiomed.com/news/articleView.html?idxno=2098. Accessed 6 Aug 2019.

[13] Nakayamada S, Kubo S, Iwata S, Tanaka Y (2016). Recent progress in JAK inhibitors for the treatment of rheumatoid arthritis. BioDrugs..

[14] Ito M, Yamazaki S, Yamagami K, Kuno M, Morita Y, Okuma K (2017). A novel JAK inhibitor, peficitinib, demonstrates potent efficacy in a rat adjuvant-induced arthritis model. J Pharmacol Sci.

[15] Takeuchi T, Tanaka Y, Iwasaki M, Ishikura H, Saeki S, Kaneko Y (2016). Efficacy and safety of the oral Janus kinase inhibitor peficitinib (ASP015K) monotherapy in patients with moderate to severe rheumatoid arthritis in Japan: a 12-week, randomised, double-blind, placebo-controlled phase IIb study. Ann Rheum Dis.

[16] Kivitz AJ, Gutierrez-Urena SR, Poiley J, Genovese MC, Kristy R, Shay K (2017). Peficitinib, a JAK inhibitor, in the treatment of moderate-to-severe rheumatoid arthritis in patients with an inadequate response to methotrexate. Arthritis Rheumatol.

[17] Tanaka Y, Takeuchi T, Tanaka S, Kawakami A, Iwasaki M, Song YW, et al. Efficacy and safety of peficitinib (ASP015K) in patients with rheumatoid arthritis and an inadequate response to conventional DMARDs: a randomised, double-blind, placebo-controlled phase III trial (RAJ3). Ann Rheum Dis. 2019;In press:annrheumdis-2019-215163. 10.1136/annrheumdis-2019-215163 PMC6788921 31350270

[18] Takeuchi T, Tanaka Y, Tanaka S, Kawakami A, Iwasaki M, Katayama K, et al. Efficacy and safety of peficitinib (ASP015K) in patients with rheumatoid arthritis and an inadequate response to methotrexate: results of a phase III randomised, double-blind, placebo-controlled trial (RAJ4) in Japan. Ann Rheum Dis. 2019;In press:annrheumdis-2019-215164. 10.1136/annrheumdis-2019-215164 PMC6788880 31350269

[19] Smolen JS, Genovese MC, Takeuchi T, Hyslop DL, Macias WL, Rooney T (2019). Safety profile of baricitinib in patients with active rheumatoid arthritis with over 2 years median time in treatment. J Rheumatol.

[20] Cohen SB, Tanaka Y, Mariette X, Curtis JR, Lee EB, Nash P, et al. Long-term safety of tofacitinib for the treatment of rheumatoid arthritis up to 8.5 years: Integrated analysis of data from the global clinical trials. Ann Rheum Dis. 2017;76:1253–62. 10.1136/annrheumdis-2016-210457 PMC5530353 28143815

[21] Winthrop KL, Yamanaka H, Valdez H, Mortensen E, Chew R, Krishnaswami S (2014). Herpes zoster and Tofacitinib therapy in patients with rheumatoid arthritis. Arthritis Rheumatol.

[22] Harigai M, Takeuchi T, Smolen JS, Winthrop KL, Nishikawa A, Rooney TP, et al. Safety profile of baricitinib in Japanese patients with active rheumatoid arthritis with over 1.6 years median time in treatment: An integrated analysis of Phase 2 and 3 trials. Mod Rheumatol. 2020;30:36–43. 10.1080/14397595.2019.1583711 30784354

[23] U.S. Food and Drug Administration. Safety trial finds risk of blood clots in the lungs and death with higher dose of tofacitinib (Xeljanz, Xeljanz XR) in rheumatoid arthritis patients; FDA to investigate. Saf. Announc. 2019. https://www.fda.gov/Drugs/DrugSafety/ucm631871.htm. Accessed 11 Mar 2019.

[24] Fleischmann R, Wollenhaupt J, Takiya L, Maniccia A, Kwok K, Wang L (2017). Safety and maintenance of response for tofacitinib monotherapy and combination therapy in rheumatoid arthritis: an analysis of pooled data from open-label long-term extension studies. RMD Open.

[25] Keystone EC, Genovese MC, Schlichting DE, De La Torre I, Beattie SD, Rooney TP (2018). Safety and efficacy of baricitinib through 128 weeks in an open-label, longterm extension study in patients with rheumatoid arthritis. J Rheumatol.

[26] Takeuchi T, Kameda H (2010). The Japanese experience with biologic therapies for rheumatoid arthritis. Nat Rev Rheumatol.

