# Correction to: Safety and effectiveness of peficitinib (ASP015K) in patients with rheumatoid arthritis: interim data (22.7 months mean peficitinib treatment) from a long-term, open-label extension study in Japan, Korea, and Taiwan

**DOI:** 10.1186/s13075-020-02247-3

**Published:** 2020-06-23

**Authors:** Tsutomu Takeuchi, Yoshiya Tanaka, Sakae Tanaka, Atsushi Kawakami, Yeong-Wook Song, Yi-Hsing Chen, Mitsuhiro Rokuda, Hiroyuki Izutsu, Satoshi Ushijima, Yuichiro Kaneko, Yoshihiro Nakashima, Teruaki Shiomi, Emi Yamada

**Affiliations:** 1 Keio University School of Medicine, Tokyo, Japan; 2 University of Occupational and Environmental Health, Japan, Kitakyushu, Japan; 3 University of Tokyo, Tokyo, Japan; 4 Nagasaki University Graduate School of Biomedical Sciences, Nagasaki, Japan; 5 Seoul National University, Seoul National University Hospital, Seoul, South Korea; 6 Taichung Veterans General Hospital, Taichung, Taiwan; 7 Astellas Pharma, Inc., Tokyo, Japan

**Correction to: Arthritis Research & Therapy (2020) 22:47**

**https://doi.org/10.1186/s13075-020-2125-2**

Following publication of the original article [1], the authors identified an error in the 95% CI bars plotted on the graphs of Fig. 6a, b, and c. The CI numbers in the table below the graph are correct, but the upper and lower limit bars plotted on the graph are incorrect. The corrected Fig. 6 is given below.

**Fig. 6:**
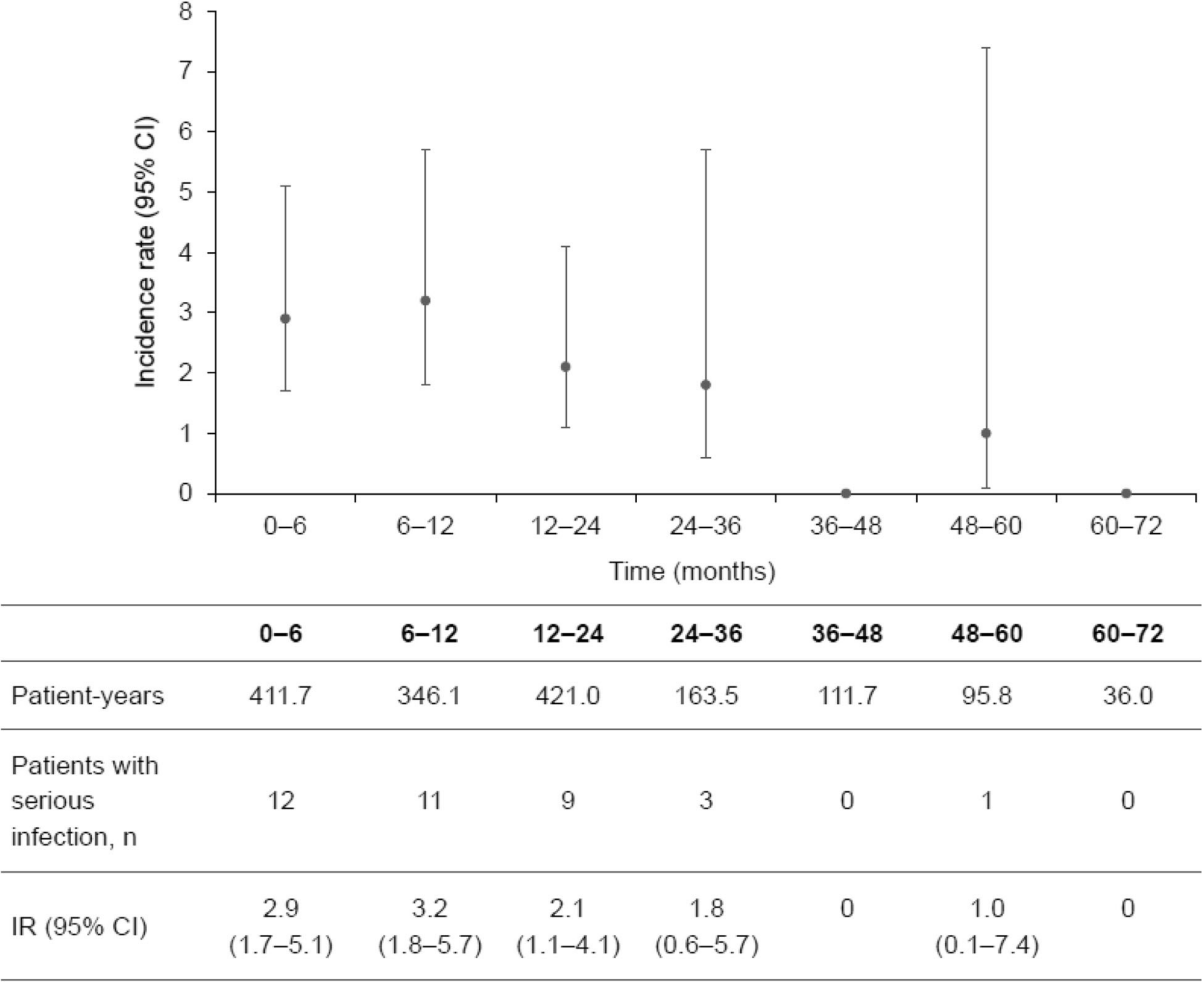
Adverse events of special interest per 100 patient-years during the overall period: **a** serious infections, **b** herpes zoster-related disease, **c** malignancies (SAF). Patient-years was calculated from initial dose up to first incidence of the event for patients who had at least one event, and from initial dose through follow up for patients who had no events; IR was calculated as (100 × number of patients with ≥ 1 incidence/total patient-years) *CI* confidence interval, *IR* incidence rate
