# Supplementary material for: Safety and effectiveness of peficitinib (ASP015K) in patients with rheumatoid arthritis: interim data (22.7 months mean peficitinib treatment) from a long-term, open-label extension study in Japan, Korea, and Taiwan
Source: Arthritis Res Ther. 2020 Mar 12;22:47. doi: 10.1186/s13075-020-2125-2 (PMC7068874; doi:10.1186/s13075-020-2125-2)
Supplement: Supplementary file 1 — Table S1 Patient demographics and characteristics by preceding study at the extension study baseline (SAF). Table S2 Peficitinib treatment exposure and changes in peficitinib dose during the overall period, by preceding study (SAF). Fig. S1 Patient flow through the study, by preceding study. Fig. S2 Response rates by preceding study: a ACR20, b ACR50, c ACR70 over time (FAS). Fig. S3 Mean changes from the baselines of the preceding studies in a TJC68, b SJC66, c SGAP, d SGA, e PGA, and f HAQ-DI (FAS). Fig. S4a Mean changes from the baselines of the preceding studies in DAS28-CRP, by preceding study; b proportion of patients achieving DAS28-CRP < 2.6, by preceding study (FAS). Study sites.Case histories of patients who died. [file 13075_2020_2125_MOESM1_ESM.docx]

# Supplementary Tables

## Table S1. Patient demographics and characteristics by preceding study at the extension study baseline (SAF)

|  | **RAJ1**  **(N=201)** | **RAJ3**  **(N=225)** | **RAJ4**  **(N=417)** |
| --- | --- | --- | --- |
| Age, years | 52.7 (11.3) | 55.3 (12.7) | 57.3 (11.4) |
| Female, n (%) | 158 (78.6) | 164 (72.9) | 297 (71.2) |
| Study region, n (%)  Japan  Korea  Taiwan | 201 (100.0)  0  0 | 188 (83.6)  19 (8.4)  18 (8.0) | 417 (100.0)  0  0 |
| RA duration at baseline of preceding study, years^1^ | 7.3 (6.1) | 8.7 (7.2) | 4.3 (3.0) |
| Patients in prednisolone dose category, n (%)  None  Average 0–5 mg/day  Average >5 mg/day | 87 (43.3)  91 (45.3)  23 (11.4) | 130 (57.8)  83 (36.9)  12 (5.3) | 215 (51.6)  167 (40.0)  35 (8.4) |
| Prior non-biological DMARD use^2^ | 143 (71.1) | 182 (80.9) | 238 (57.1) |
| Prior biological DMARD-IR^3^ | 18 (9.0) | 16 (7.1) | 1 (0.2) |
| Patients receiving concomitant DMARD, n (%)  None  MTX  DMARD except for MTX | 201 (100)  0  0 | 29 (12.9)  136 (60.4)  60 (26.7) | 4 (1.0)  413 (99.0)  0 |
| Maximum MTX dose category, n (%)  None  0–≤8 mg/week  8–≤12 mg/week  >12 mg/week | 0  0  0  0 | 91 (40.4)  44 (19.6)  58 (25.8)  32 (14.2) | 6 (1.4)  205 (49.2)  162 (38.8)  44 (10.6) |
| Tender joint count at 68 joints^4^ | 10.8 (9.6) | 3.2 (5.1) | 3.4 (6.0) |
| Swollen joint count at 66 joints^4^ | 8.2 (6.8) | 2.8 (4.2) | 2.6 (3.6) |
| HAQ-DI score^5^ | 0.86 (0.62) | 0.50 (0.52) | 0.51 (0.55) |
| CRP, mg/dL^6^ | 1.89 (2.28) | 0.58 (1.02) | 0.54 (0.96) |
| ESR, mm/h^6^ | 42.3 (24.3) | 24.3 (20.4) | 24.0 (20.1) |
| DAS28-CRP^4^ | 4.53 (1.42) | 2.62 (1.08) | 2.53 (1.14) |
| DAS28-ESR^4^ | 5.20 (1.44) | 3.19 (1.24) | 3.11 (1.28) |
| CDAI score^4^ | 23.44 (14.37) | 8.18 (7.57) | 7.75 (8.04) |
| SDAI score^4^ | 25.33 (15.46) | 8.75 (8.01) | 8.31 (8.48) |

Data are expressed as mean (SD) unless otherwise stated.

^1^Duration of RA was calculated as (date of screening visit of preceding study – onset date of RA + 1)/365.25.

^2^Except for MTX use, at baseline of preceding study.

^3^At baseline of preceding study.

^4^Higher scores indicate greater levels of disease activity.

^5^Possible HAQ-DI scores range 0–3, with higher scores indicating greater disability.

^6^Higher CRP and ESR values indicate greater inflammation.

CDAI, Clinical Disease Activity Index; CRP, C-reactive protein; DAS, Disease Activity Score; DMARD, disease-modifying antirheumatic drug; ESR, erythrocyte sedimentation rate; HAQ-DI, Health Assessment Questionnaire – Disability Index; IR, inadequate response; MTX, methotrexate; RA, rheumatoid arthritis; SAF, safety analysis set; SDAI, Simplified Disease Activity Index.

## Table S2. Peficitinib treatment exposure and changes in peficitinib dose during the overall period, by preceding study (SAF)

|  | **RAJ1**  **(N=201)** | **RAJ3**  **(N=225)** | **RAJ4**  **(N=417)** |
| --- | --- | --- | --- |
| Duration of peficitinib exposure, months^1^  Mean (SD)  Max  Median  Min | 41.6 (24.6)  70.7  51.1  0.5 | 17.9 (7.3)  32.1  18.4  0.9 | 16.2 (8.0)  33.1  15.3  0.1 |
| Duration of initial peficitinib dose, months^2^  Mean (SD)  Max  Min | 10.4 (18.1)  70.7  0.5 | 13.1 (8.6)  32.0  0.9 | 12.4 (8.7)  32.9  0.1 |
| Treatment compliance rate (%)^3^  Mean (SD) | 98.8 (1.9) | 96.3 (4.5) | 96.4 (3.2) |
| Dose increase, n (%)  No  Yes  1 dose increase  2 dose increases  ≥3 dose increases | 39 (19.4)  162 (80.6)  102 (50.7)  56 (27.9)  4 (2.0) | 156 (69.3)  69 (30.7)  64 (28.4)  5 (2.2)  0 | 294 (70.5)  123 (29.5)  119 (28.5)  2 (0.5)  2 (0.5) |
| Dose decrease, n (%)  No  Yes  1 dose decrease  2 dose decreases  ≥3 dose decreases | 192 (95.5)  9 (4.5)  7 (3.5)  2 (1.0)  0 | 210 (93.3)  15 (6.7)  14 (6.2)  1 (0.4)  0 | 400 (95.9)  17 (4.1)  17 (4.1)  0  0 |
| Maximum peficitinib dose, n (%)  50 mg  100 mg  150 mg | 39 (19.4)  104 (51.7)  58 (28.9) | 0  160 (71.1)  65 (28.9) | 0  297 (71.2)  120 (28.8) |

^1^Duration of exposure for overall period (days) was calculated as: date of the last dose of study drug – date of initial dose of study drug + 1.

^2^Duration from first peficitinib taken (50 mg for patients from RAJ1, 100 mg for patients from RAJ3 and RAJ4) up to first dose change was calculated.

^3^Treatment compliance for overall period (%) was calculated as: 100 × (total number of tablets actually received in the overall period / total number of tablets planned to receive in the overall period).

SAF, safety analysis set; SD, standard deviation.

Supplementary Figures

## Fig. S1 Patient flow through the study, by preceding study


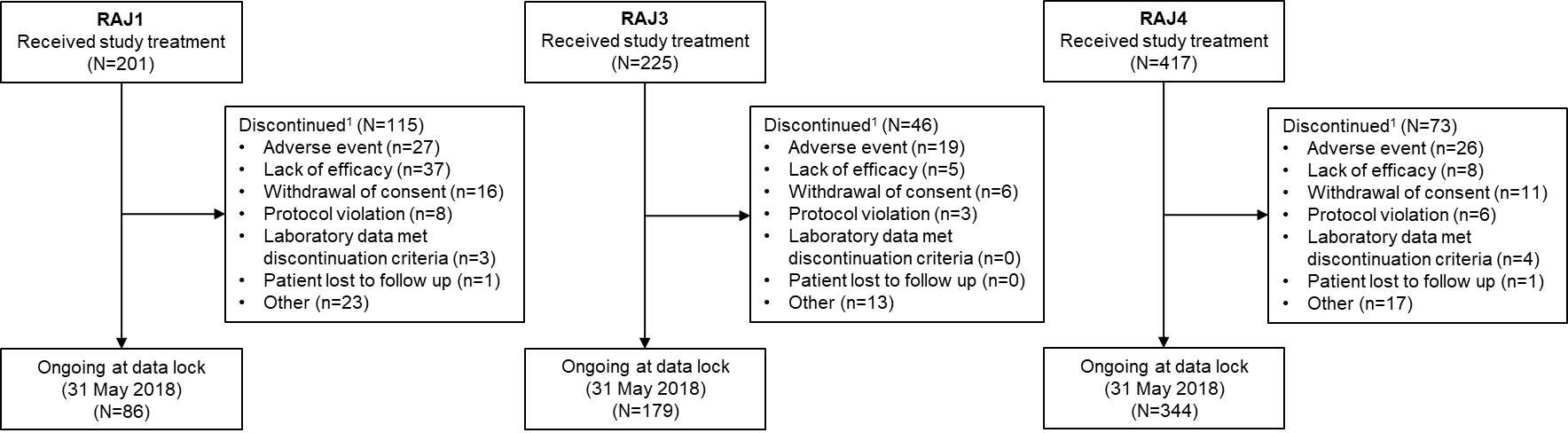


^1^Discontinuation for overall period: discontinued at any time from start of initial dosing of study drug through the last dose day for overall period.

## Fig. S2 Response rates by preceding study: (a) ACR20; (b) ACR50; (c) ACR70 over time (FAS)

(a) ACR20


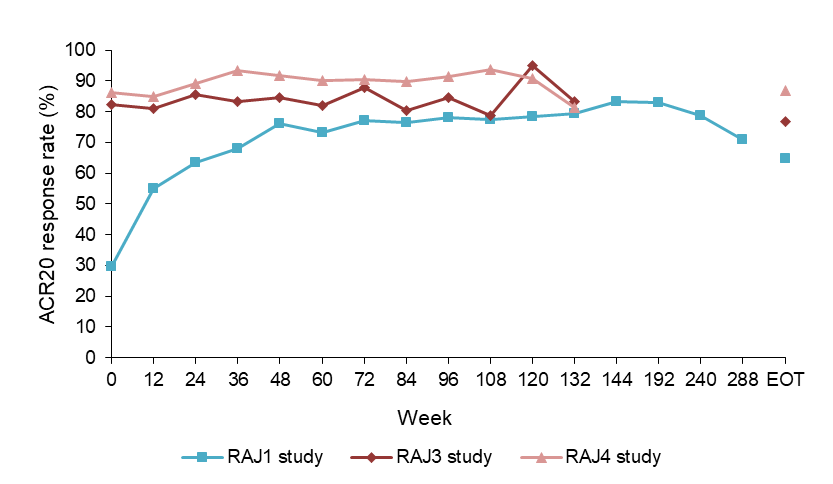


(b) ACR50


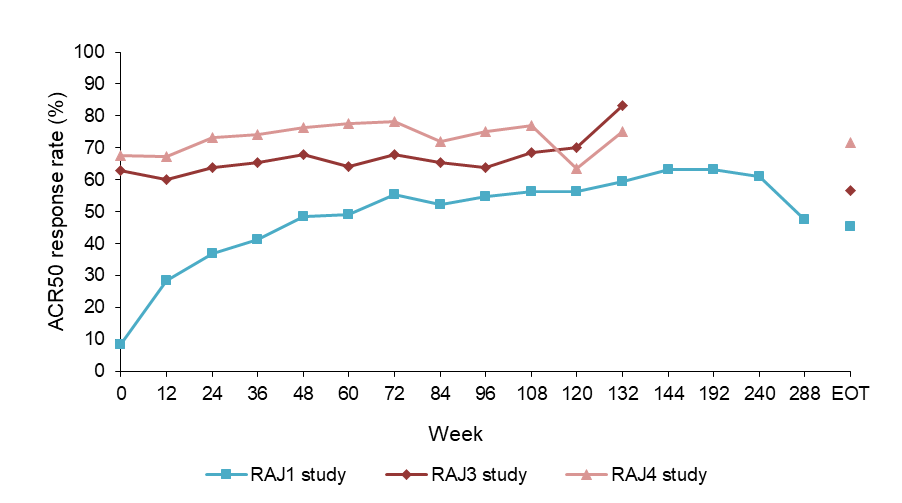


(c) ACR70


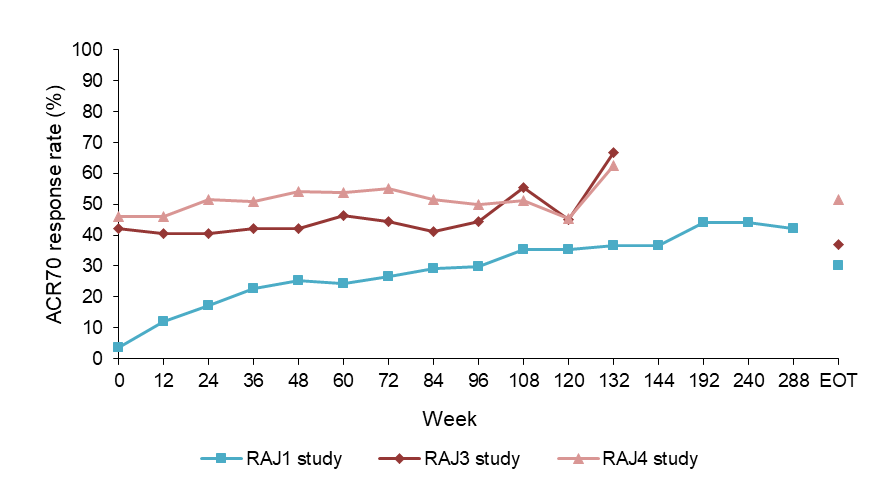


## Fig. S3 Mean changes from the baselines of the preceding studies in (a) TJC68; (b) SJC66; (c) SGAP; (d) SGA; (e) PGA; and (f) HAQ-DI (FAS)

1. TJC68


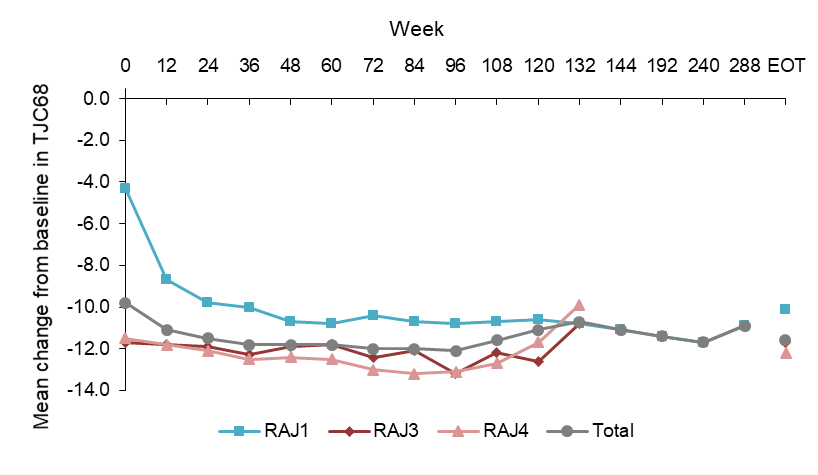


1. SJC66


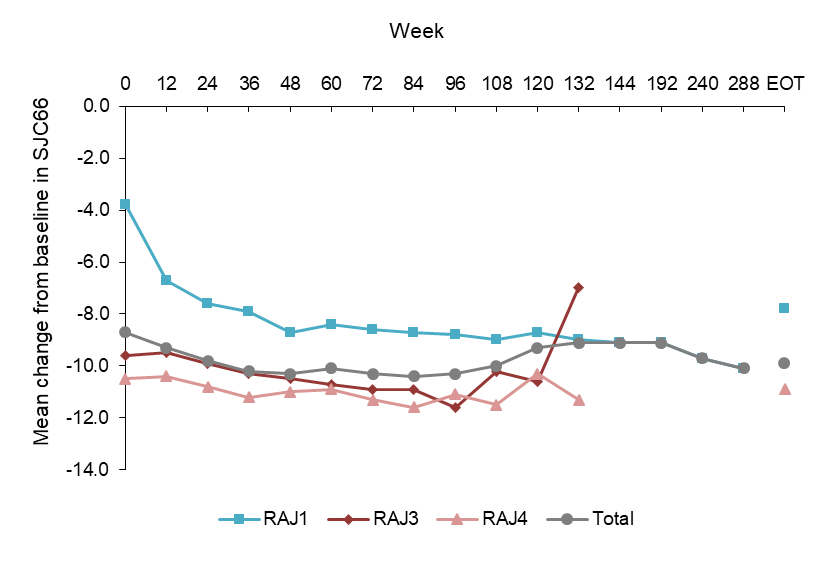


(c) SGAP (100 mm VAS)


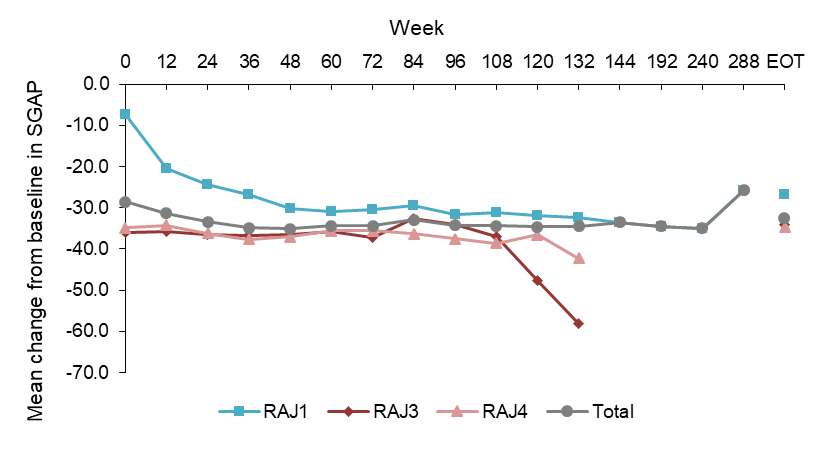


(d) SGA (100 mm VAS)


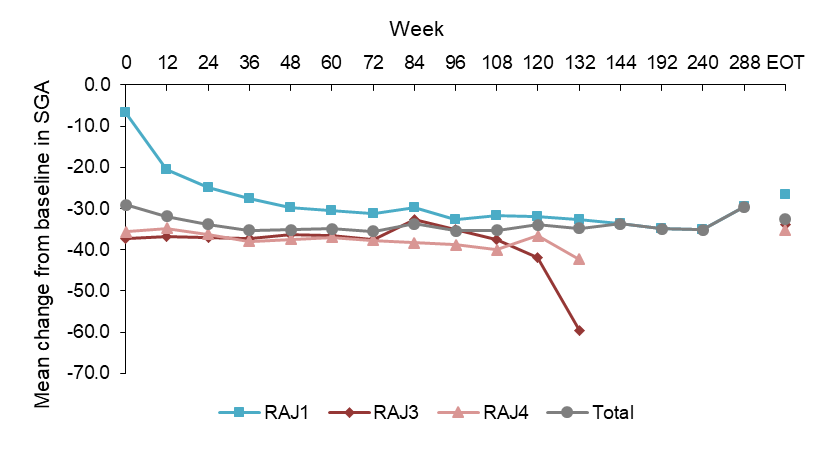


(e) PGA (100 mm VAS)


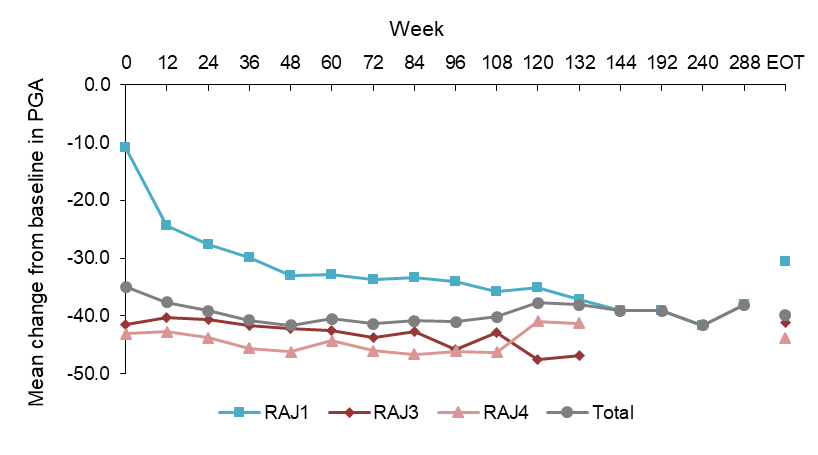


(f) HAQ-DI


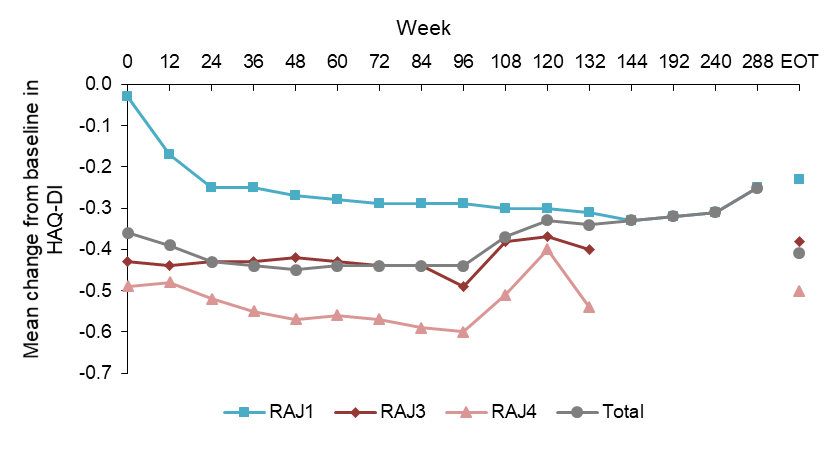


## Fig. S4 (a) Mean changes from the baselines of the preceding studies in DAS28-CRP, by preceding study; (b) Proportion of patients achieving DAS28-CRP <2.6, by preceding study (FAS)

(a) DAS28-CRP


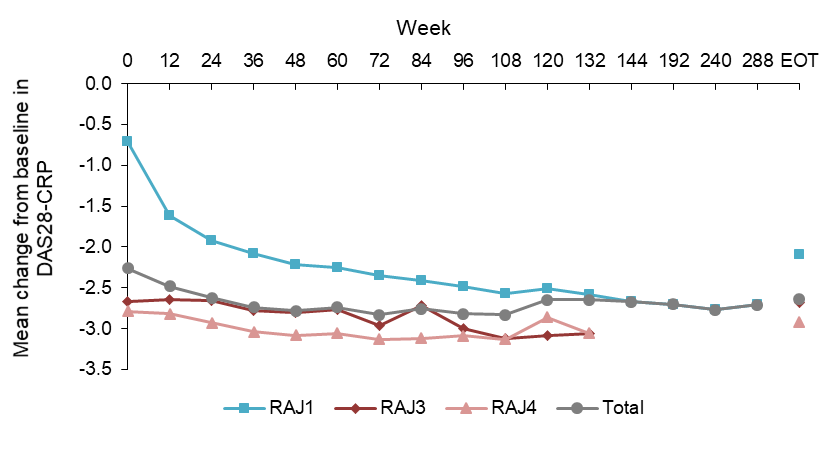


(b) Proportion of patients achieving DAS28-CRP <2.6


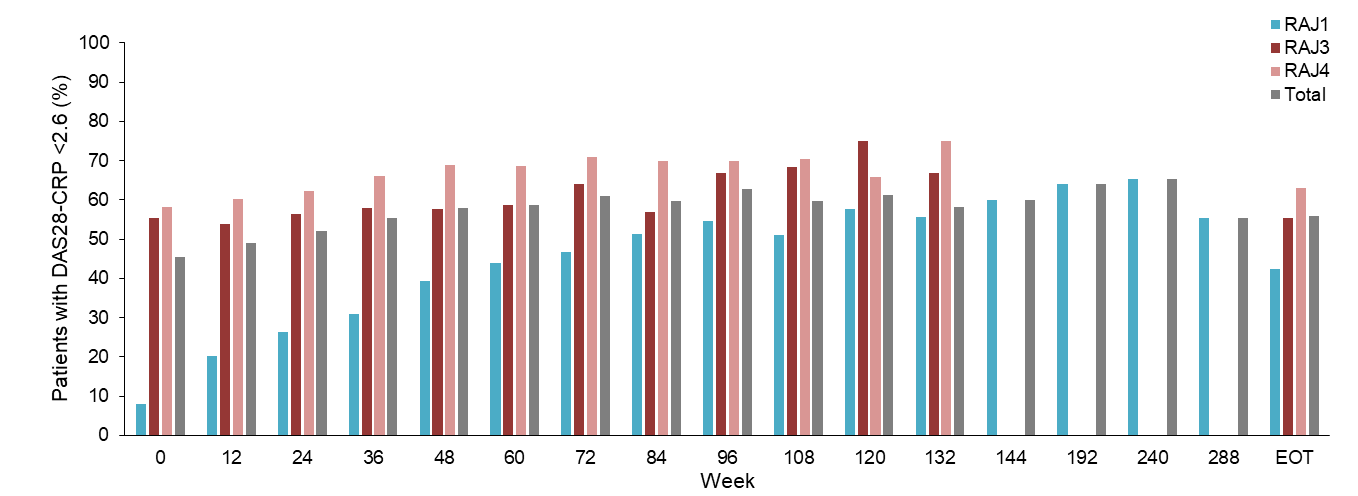


Supplementary documents

## Study sites

**Japan:** Ohsaki Medicine; Sagawa Akira Rheumatology Clinic; Sapporo City General Hospital; Hikarigaoka Spellman Hospital; Dainohara Orthopedics; Nephrology and Rheumatology, Niigata University Medical and Dental Hospital; Ohno Clinic; Hirose Clinic; Matuno Clinic for Rheumatic Diseases; Honjo Rheumatism Clinic; Osaka Rehabilitation Hospital; Kobe Konan Yamate Clinic; Hiroshima Red Cross Hospital & Atomic-bomb Survivors Hospital; Hiroshima Rheumatology Clinic; Shigenobu Orthopedics Rheumatism Rehabilitation Clinic; Ishioka Clinic; Oribe Clinic of Rheumatology and Internal Medicine; Shono Rheumatism Clinic; Kumamoto Saishunso National Hospital; Internal Medicine and Rheumatology, National Hospital Organization Kyushu Medical Center; Uchida Clinic; Kumamoto Orthopaedic Hospital; Yu Family Clinic; Tokyo Medical and Dental University Hospital; Nagaoka Red Cross Hospital; Katayama Orthopedic Rheumatology Clinic; Higashisendai Rheumatology Medical Clinic; Yoshida Clinic Orthopedic Surgery and Rheumatology; Niigata University Medical and Dental Hospital; Nishinarita Clinic Office; Hokkaido University Hospital; Institute of Rheumatology Tokyo Women's Medical University; Inoue Hospital; Komatsu Municipal Hospital; Kuroda Orthopaedic Hospital; Aoikai Medical Corporation Sendai Taihaku Hospital; Nagoya University Hospital; Hokkaido Medical Center for Rheumatic Diseases; University of Tsukuba Hospital; University of Occupational and Environmental Health Hospital; Matsubara Mayflower Hospital; Matsubara clinic; Allergy and Rheumatology, The University of Tokyo Hospital; Miyake Orthopedic Clinic; Kan Rheumatism And Orthopedics Clinic; Kumamoto Rheumatology Clinic, Miyasato Clinic; Kawasaki Municipal Hospital; Komagamine Rheumatic Orthopaedic Clinic, Ohira Orthopaedic Hospital; Osafune clinic; Azuma Rheumatology Clinic; Sugimoto Clinic; Mito Saiseikai General Hospital; Hiroshima Clinic; Okubo Hospital; Tsurukami Clinic of Orthopaedic and Rheumatology; Kawasaki RA& IM Clinic; Rheumatology, National Hospital Organization Kyushu Medical Center; Ogawa Internal Medical Clinic; Toneyama National Hospital; Kaneko internal medicine rheumatology clinic; Fujimori Clinic; National Hospital Organization Beppu Medical Center; Eiraku Internal Medicine Clinic; Suzuki Clinic, National Hospital Organization Fukuoka Hospital; Kainan Hospital; Himeji Medical Center; Saiseikai Suita Hospital; St. Mary's Hospital; National Hospital Organization Tokyo Medical Center; Japanese Red Cross Koga Hospital; Kagoshima Red Cross Hospital; National Hospital Organization Ureshino Medical Center; Kyushu Central Hospital; Kamitsuga General Hospital; National Hospital Organization Osaka Minami Medical Center; Nagano Municipal Hospital; Japanese Red Cross Medical Center; Toho University Ohashi Medical Center; Tokai University Hospital; University Hospital Kyoto Prefectural University of Medicine; Orthopaedic Surgery_Osaka University Hospital; Kagawa University Hospital; Shizuoka Riumachi Seikeigeka Rehabili Hospital; Hakodate Goryoukaku Hospital; Orthopaedic Surgery, Kobe University Hospital; Bay Side Misato Medical Center; Higami Hospital, Mitsui Memorial Hospital, Yokohama City Minato Red Cross Hospital; Nagasaki Medical Hospital of Rheumatology; Sasaki Foundation Kyoundo Hospital; Matsuta Clinic; National Hospital Organization Nagasaki Medical Center; Kamagaya General Hospital; Miyashita Rheumatology Clinic; Tokito Clinic Rheumatology & Orthopaedic Surgery; Northern Capital Clinic; Inokuchi Clinic; Oasis Clinic; Toyohashi Medical Center; National Hospital Organization Nagoya Medical Center; The Japanese Red Cross Nagasaki Genbaku Hospital; Tonan Hospital; Chibaken Saiseikai Narashino Hospital; Hyogo College Of Medicine College Hospital; Japanese Red Cross Okayama Hospital; Shinkokura Hospital; Iizuka Hospital; Zenjinkai Shimin-No-mori Hospital; Japanese Red Cross Kyoto Daiichi Hospital; OKI Medical Clinic; Kushiro Red Cross Hospital; Med.Corp.Sokokai Affiliated Clinic of Gyoda General Hospital; Marunouchi Hospital; Chubu Rosai Hospital; Hamanomachi Hospital; Chutoen General Medical Center; Higashiosaka city medical center; Japanese Red Cross Shizuoka Hospital; Kinki Central Hospital; Kindai University Sakai Hospital; National Hospital Organization, Shimoshizu National Hospital; Toyama University Hospital; Fujita Health University Hospital; National Hospital Organization Sagamihara Hospital; St. Luke's International Hospital; Orthopaedic Surgery and Spinal Surgery, The University of Tokyo Hospital; Niigata Rheumatic Center; Jichi Medical University Hospital; Clinical Immunology, Osaka University Hospital; Nagasaki University Hospital; Toho University Omori Medical Center; Juntendo University Hospital; Osaka City University Hospital; Tohoku University Hospital; Nippon Medical School Hospital; Sasebo Chuo Hospital; Rheumatology and Clinical Immunology_Kobe University Hospital; Shirahama Hamayu Hospital; Toyota Kosei Hospital; Osaka Rheumatology Clinic; Sapporo Medical Center; NTT East Corporation; Kyoto University Hospital; Saitama Medical Center; JCHO Isahaya General Hospital; Higashihiroshima Memorial Hospital; Kyushu University Hospital; Fukushima Daiichi Hospital; Funabashi Municipal Medical Center; Medical corporation Nagamine orthopedics Clinic; JCHO Yokkaichi Hazu Medical Center; Osaki Citizen Hospital; Toyohashi Municipal Hospital; Shinko Hospital; Japanese Red Cross Kitami Hospital; Nagano Red Cross Hospital; Daido Clinic; Sugimoto Rheumatology and Internal Medicine Clinic; Miyashima RA & Orthopaedic Clinic; Morita Hospital; Asahi General Hospital; Hitachi Ltd. Hitachinaka General Hospital.

**Korea:** Seoul National University Hospital; Hanyang University Seoul Hospital; Daegu Catholic University Medical Center; Chonnam National University Hospital; Inha University Hospital; Ajou University Hospital; Chonbuk National University Hospital; KonKuk University Hospital; KyungHee University Hospital.

**Taiwan:** National Taiwan University Hospital; Taipei Veterans General Hospital; Chang Gung Memorial Hospital-LinKou; Taichung Veterans General Hospital; China Medical University Hospital; Kaohsiung Veterans General Hospital; Chung Shan Medical University Hospital; Cathay General Hospital; National Cheng Kung University Hospital.

## Case histories of patients who died

### Death due to malignant lymphoma during the study

A 65-year-old man from Japan enrolled in the RAJ4 study and started placebo treatment on 09 April 2015. He switched to peficitinib 100 mg/day at Week 12 on 02 July 2015. On 07 April 2016 (day 1), the patient provided informed consent for the RAJ2 study and started treatment with peficitinib 100 mg/day. The patient had a medical history of diabetes, hyperuricemia, hyperlipidemia, right kidney cyst, constipation, colon polyp, colon diverticulosis, trichophytosis unguium and pulmonary emphysema. He was also receiving concomitant methotrexate (oral, 12 mg/week). Other concomitant medications at the time of the first dose of peficitinib in the extension study included prednisolone, teprenone, loxoprofen sodium, magnesium oxide, folic acid, risedronate sodium, ketoprofen, alogliptin benzoate, febuxostat, glimepiride, metformin hydrochloride, atorvastatin calcium, omega-3-acid ethyl ester, adenosine triphosphate, disodium salt and betahistine mesilate.

On 17 June 2016 (day 72), the patient began to experience thoracoabdominal pain, appetite impairment, excessive sweating and malaise. On 21 June 2016 (day 76), the patient was admitted to hospital with pyrexia, elevated CRP (9.55 mg/dL) and elevated lactate dehydrogenase (3365 U/L). Thoracoabdominal CT examination showed multiple hepatic and splenic masses, with a suspected diagnosis of malignant lymphoma. Treatment with peficitinib and MTX were suspended on 21 June 2016. On 24 June 2016, bone marrow aspiration confirmed the diagnosis of malignant lymphoma and the investigator discontinued the study. Disseminated intravascular coagulation developed, associated with the malignant lymphoma.

On 25 June 2016 (day 80), chemotherapy with intravenous etoposide, intravenous vincristine sulfate and intravenous doxorubicin hydrochloride was initiated, but was interrupted later the same day following observation of pupillary inequality and a depressed level of consciousness. CT examination revealed a subdural hematoma, which was evacuated by craniotomy. On 26 June 2016 (day 81), post-operative pupillary inequality improved after artificial respiration control, transfusion and DIC treatment were administered. On 27 June 2016 (day 82), hemorrhage was observed in the left subdural space. Chemotherapy was resumed on 29 June 2016 (day 84), and convulsions began to occur on the same day. On 30 June 2016 (day 85), SpO_2_ decreased to 80–90%, and further convulsions, pupillary inequality and loss of light reflex in the right pupil were observed. CT and MRI examinations showed a midline shift and brain infarction. Chemotherapy was terminated on 03 July 2016 (day 88). On 04 July 2016 (day 89), aspiration was performed, SpO2 and heart rate decreased, and dilated pupils and loss of brainstem reflex were observed. During the morning of 06 July 2016 (day 91), the patient’s respiratory status was unstable, and cyanosis and cold feeling were noted. On 07 July 2016 (day 92), the patient was diagnosed with advanced neutropenia associated with chemotherapy, left atelectasis due to sputum retention, hepatic shock and renal failure. A blood culture test showed gram-negative rods. On 08 July 2016 (day 93), heart rate decreased to 40–50 bpm and intermittent convulsion developed. The patient died on 09 July 2016 (day 94).

The investigator considered the malignant diffuse large B-cell lymphoma probably related to the study drug as the event developed after initiation of peficitinib, although the event could also be due to the underlying disease of long-term MTX treatment.

### Death due to uterine sarcoma after discontinuation from the study

A 65-year-old woman from Japan who was enrolled in the RAJ1 study received her first dose of peficitinib 150 mg/day on 11 May 2012. The patient had a medical history of hyperlipidemia, osteoporosis, urine occult blood reaction and hypertension. Concomitant medications at the time of first dose of peficitinib in the extension study included atorvastatin calcium, risedronate sodium, amlodipine besilate, methylprednisolone, nabumetone and rebamipide. Following enrolment in the RAJ2 study, the patient started to receive peficitinib 50 mg/day on 31 August 2012 (day 1).

On 12 April 2013 (day 225), mild elevation of CRP and leukocyte count were noted, although there were no signs of infection; on 07 June 2013, the study drug was discontinued following further increases in CRP and leukocyte count. On 20 July 2013 (day 324), positron emission tomography (PET)/computed tomography (CT) showed a suspected uterine cancer. Further examination on 26 July 2013 (day 330) found a suspected myoma (size 4.9 cm) and endometritis. On 12 August 2012 (day 347), a transvaginal scan revealed a 9.9 cm-sized tumor. Further assessment on 30 September 2013 (day 396) and 11 October 2013 (day 407) indicated gastrointestinal perforation, symptoms of ileus and adherence of the uterine tumors to the bladder and rectum. Uterine sarcoma or cancer was diagnosed. As the patient was unable to take meals, she experienced malnutrition and dehydration. She was discharged from hospital care on 23 October 2013 (day 419).

In December 2013, the patient presented with edema, vomiting, and abdominal pain; there were also signs of hematuria. On 13 December 2013 (day 470), the patient began to receive drip infusion treatment with high-calorie infusion, prochlorperazine maleate, oxycodone hydrochloride, octreotide acetate, rebamipide, acetaminophen and fentanyl citrate. Draining of the ascites was also performed. An oxygen tube and ureteral catheter were placed. From 23 December 2013 (day 483), the patient was poorly responsive to verbal stimulation, and all oral drugs were discontinued. The patient became dependent on drip infusion for nutritional support from 26 December 2013 (day 483), and died on 28 December 2013 (day 485).

Other adverse events experienced by the patient during the study included chronic gastritis and sliding hiatal hernia. The investigator considered the elevation of CRP and leukocytes possibly related to peficitinib, and the suspected uterine sarcoma possibly related to peficitinib, as the tumor may have developed after initiation of the study drug.
